# Influenza D Virus of New Phylogenetic Lineage, Japan

**DOI:** 10.3201/eid2601.191092

**Published:** 2020-01

**Authors:** Shin Murakami, Ryota Sato, Hiroho Ishida, Misa Katayama, Akiko Takenaka-Uema, Taisuke Horimoto

**Affiliations:** University of Tokyo, Tokyo, Japan (S. Murakami, H. Ishida, M. Katayama, A. Takenaka-Uema, T. Horimoto);; Yamagata Livestock Hygiene Service Center, Yamagata, Japan (R. Sato)

**Keywords:** influenza D virus, bovine, heterologous, lineage, respiratory infections, Japan, influenza, viruses

## Abstract

Influenza D virus (IDV) can potentially cause respiratory diseases in livestock. We isolated a new IDV strain from diseased cattle in Japan; this strain is phylogenetically and antigenically distinguished from the previously described IDVs.

Influenza D virus (IDV; family *Orthomyxoviridae*) is one of the possible bovine respiratory disease complex (BRDC) causative agents. IDVs are detected in and isolated from cattle in many countries in North America, Asia, Europe, and Africa (*1**–**4*). In addition, both IDV RNAs and specific antibodies have been detected in many animal species (*1**,**5**,**6*). To date, IDVs have been phylogenetically classified into 3 lineages: D/OK, D/660, and Japanese lineages. We isolated a new IDV strain from cattle in Japan with respiratory disease, whose hemagglutinin-esterase-fusion (HEF) gene did not belong to the known phylogenetical lineages.

At a herd in Yamagata Prefecture in northern Japan, 15 Holsteins (37.5% of bred cattle in the herd) had respiratory signs develop during January 6–10, 2019 (Appendix Table 1). We collected nasal swab samples from 9 of 15 cows on January 7 and 10. All samples subjected to IDV-specific real-time reverse transcription PCR (RT-PCR) (*7*) were found positive. We also subjected these samples to RT-PCR analyses specific for bovine viral diarrhea virus 1 and 2, infectious bovine rhinotracheitis virus, bovine parainfluenza virus 3, bovine respiratory syncytial virus, bovine coronavirus, bovine rhinitis A virus, *Mycobacterium bovis*, *Mycoplasma bovigenitalium*, *Mycoplasma dispar*, *Ureaplasma diversum*, and *Mycoplasma bovirhinis*. All samples were found negative for these pathogens except for the sample from cow no. 5, which was positive for *M. dispar*. Despite these cows receiving antimicrobial drug treatment, we isolated *Mannheimia haemolytica* from 7 of them (nos. 1–5, 7, 8); cow no. 5 died on January 7. Taken together, these factors suggested that IDV infection was involved in the onset of BRDC in this herd.

To isolate IDV, we inoculated the collected samples into human rectal tumor HRT-18G cells (American Type Culture Collection [ATCC] no. CRL-11663), incubated the cells for 4 days at 37°C, and blindly passaged the supernatants in swine testis ST cells (ATCC no. CRL-1746) in the presence of tosyl phenylalanyl chloromethyl ketone-treated trypsin. We observed a cytopathic effect at the second passage. For further analysis, we used supernatants that tested positive for hemagglutination (HA) and for presence of IDV RNA detected by real-time RT-PCR, indicating successful isolation of IDV (designated as D/bovine/Yamagata/1/2019 [D/Yama2019]). We determined the entire genomic sequence of D/Yama2019 by performing RT-PCR amplification of each viral segment with specific primers, revealing an identical sequence with that detected in swab sample no. 1. We deposited our sequence data in GenBank (accession nos. LC494105–11).

We phylogenetically analyzed the sequences of D/Yama2019 by performing maximum-likelihood analysis using MEGA version X (*8*). The HEF sequence of D/Yama2019 acquired an independent position different from that exhibited in any other lineage, whereas those of the other segments were positioned relatively close to the preexisting Japanese strains, in which polymerase basic protein 1 and 2, polymerase protein 3, nucleoprotein, and nonstructural protein sequences were of the Japanese lineage, whereas the matrix sequence was of the D/OK lineage (Figure).

**Figure:**
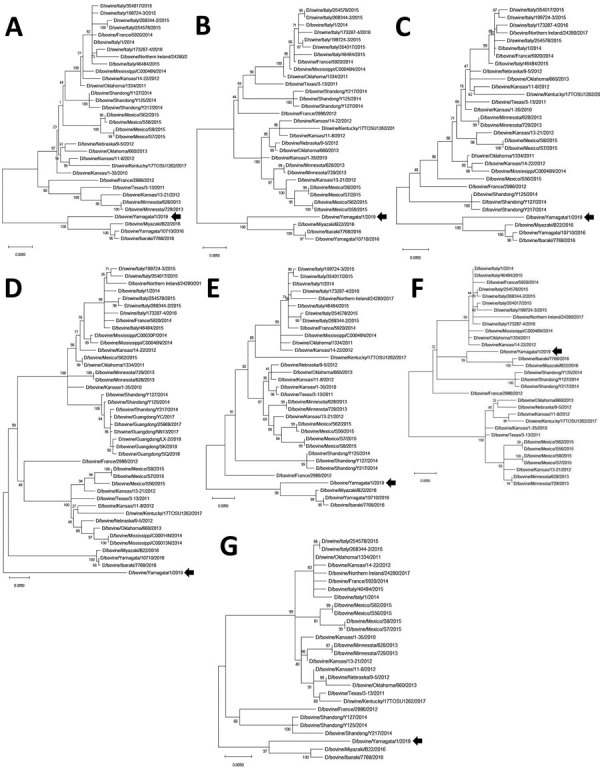
Phylogenetic trees for the 7 nucleotide genomic segments of D/bovine/Yamagata/1/2019 (arrow), an influenza D virus of a new phylogenetic lineage, Japan. A) Polymerase basic protein 2; B) polymerase basic protein 1; C) polymerase protein 3; D) hemagglutinin-esterase-fusion protein; E) nucleoprotein; F) matrix protein; and G) nonstructural protein segments. Maximum-likelihood analysis, in combination with 500 bootstrap replicates, was used to derive trees based on nucleotide sequences of the genomic segments. Bootstrap values are shown above and to the left of the major nodes. Scale bars indicate nucleotide substitutions per site.

We also collected serum samples from 8 cows on January 10 (acute phase of the disease) and February 4 (recovery phase) and examined them for antibodies against D/Yama2019 by using the HA inhibition (HI) test (Appendix Table 1). We treated serum samples with receptor-destroying enzyme (RDEII; Denka Seiken, Tokyo, Japan*,* https://www.denka.co.jp) before the HI test, which was done with the D/Yama2019 antigen and 0.6% turkey erythrocyte suspension. We considered samples with HI titer >1:40 to be positive (*4*). All serum samples collected on January 7 were HI-negative except for the sample from cow no. 1 (a titer of 1:40), whereas all samples collected on February 4 were HI-positive (a titer range of 1:40–1:160), confirming seroconversion to IDV antibodies in diseased cows. Taken together, these results indicate that cattle were infected with IDV, leading to BRDC in this herd.

We next compared HEF antigenicities among D/Yama2019 and 3 reference IDVs, D/swine/Oklahoma/1334/2011 (D/OK) in the D/OK-lineage (*1*), D/bovine/Nebraska/9–5/2012 (D/NE) in the D/660-lineage (*9*), and D/bovine/Yamagata/10710/2016 (D/Yama2016) in the Japanese-lineage (*10*), by performing HI tests using anti-IDV mouse serum and a panel of anti-HEF monoclonal antibodies (Appendix Table 2). We observed 1-way cross-reactivity between D/Yama2019 and D/OK or D/Yama2016 with antiserum. Monoclonal antibody clones B4 and R36 cross-reacted to D/Yama2019 and other tested viruses with different titers, whereas the clones G22, G27, and G74, which reacted strongly to D/Yama2016, did not react to D/Yama2019. These data indicated HEF antigenic heterogeneity between D/Yama2019 and viruses of the 3 known lineages. Monoclonal antibodies revealed the presence of common epitopes among IDVs, as suggested by previous reports (*9**,**10*). Amino acid differences were located on the head region of HEF among the IDVs, possibly causing antigenic heterogeneity.

In summary, we isolated a newly identified IDV from diseased cattle that was phylogenetically and antigenically distinguished from known IDVs. Further epidemiologic studies are needed to clarify invasion and epidemic status of these heterologous IDVs, but our results indicated that heterologous IDVs are circulating in close vicinity within the prefecture.

Appendix Further information about the study of influenza D in cattle, Japan.
