# Supplementary material for: Influenza D Virus of New Phylogenetic Lineage, Japan
Source: Emerg Infect Dis. 2020 Jan;26(1):168–71. doi: 10.3201/eid2601.191092 (PMC6924913; doi:10.3201/eid2601.191092)
Supplement: Appendix — Further information about the study of influenza D in cattle, Japan. [file 19-1092-Techapp-s1.pdf]

Article DOI: https://doi.org/10.3201/eid2601.191092

Influenza D Virus of New Phylogenetic Lineage, Japan

Appendix

Appendix Table 1. Cattle tested in the study of influenza D virus, Japan*

Cow no.	Age, mo	Clinical onset of respiratory illness	IDV qRT-PCR	HI titer	
				2019 Jan 10	2019 Feb 4
1	78	2019 Jan 6	Positive (2019 Jan 7)†	40	160
2	80	2019 Jan 6	Positive (2019 Jan 7)	<40	160
3	48	2019 Jan 6	Positive (2019 Jan 7)	<40	80
4	116	2019 Jan 7	Positive (2019 Jan 7)	<40	160
5	40	2019 Jan 6	Positive (2019 Jan 10)	ND	ND
6	43	2019 Jan 8	Positive (2019 Jan 10)	<40	160
7	53	2019 Jan 10	Positive (2019 Jan 10)	<40	160
8	29	2019 Jan 9	Positive (2019 Jan 10)	<40	160
9	69	2019 Jan 9	Positive (2019 Jan 10)	<40	40

*HI (hemagglutinin-inhibition) titers against D/bovine/Yamagata/1/2019; IDV, influenza D virus; ND, not determined; qRT-PCR, quantitative reverse transcription PCR

†Dates indicate dates on which nasal swabs were collected.

Appendix Table 2. HI titers of polyclonal and monoclonal antibodies to IDV, Japan, 2019*

	Virus			
Antibody	D/Yama2019	D/Yama2016	D/OK	D/NE
anti-D/OK serum	80	640	320	40
anti-D/Yama2016 serum	80	320	640	40
anti-D/Yama2019 serum	640	320	320	80
B4 mAb	1,280	2,560	40	2,560
B13 mAb	<40	<40	<40	<40
R36 mAb	5,120	40,960	40,960	320
G22 mAb	<40	5,120	320	40
G27 mAb	<40	>81,960	<40	<40
G74 mAb	<40	320	<40	80

*D/Yama2019, D/bovine/Yamagata/1/2019; D/Yama2016, D/bovine/Yamagata/10710/2016; D/OK, D/swine/Oklahoma/1334/2011; D/NE, D/bovine/Nebraska/9–5/2012; HI, hemagglutination-inhibition; IDV, influenza D virus; mAb, monoclonal antibody raised against D/bovine/Yamagata/10710/2016 in BALB/c mice.
